# Comparative Evaluation of the Safety and Efficacy of Long-Term Use of Imidafenacin and Solifenacin in Patients with Overactive Bladder: A Prospective, Open, Randomized, Parallel-Group Trial (the LIST Study)

**DOI:** 10.1155/2011/854697

**Published:** 2011-10-20

**Authors:** Masayoshi Zaitsu, Koji Mikami, Noriko Ishida, Takumi Takeuchi

**Affiliations:** ^1^Department of Urology, Kanto Rosai Hospital, 1-1 Kizukisumiyoshi-cho, Nakahara-ku, Kawasaki 211-8510, Japan; ^2^Department of Pharmacy, Kanto Rosai Hospital, 1-1 Kizukisumiyoshi-cho, Nakahara-ku, Kawasaki 211-8510, Japan

## Abstract

*Objectives*. Overactive bladder (OAB) is a chronic disease, but comparative trials of anticholinergics, which are commonly used for treatment of OAB, have generally been performed for up to 12 weeks only. There is no comparative study of a long-term intervention. *Methods*. We conducted a 52-week prospective randomized comparative study to evaluate the efficacy and tolerability of two anticholinergics. *Results*. Forty-one Japanese patients with untreated OAB were randomly assigned to imidafenacin and solifenacin groups. There was no difference in OABSS and KHQ scores between the two groups, but the severity and incidence of adverse events caused by the anticholinergics showed increased differences between the groups with time. The severity of dry mouth and the incidence of constipation were significantly lower in the imidafenacin group (*P* = 0.0092 and *P* = 0.0013, resp.). *Conclusions*. This study is the first long-term trial to show differences in the properties of anticholinergics that were not detected in short-term studies. Since OAB is a chronic disease, we conclude that imidafenacin is preferable to solifenacin from a perspective of safety.

## 1. Background

Overactive bladder (OAB) is a syndrome defined by symptoms of urinary urgency with or without urge incontinence, and usually with frequency and nocturia [1]. The incidence of OAB ranges from 10% to 20% in Europe and the United States [2, 3]. The incidence in people aged ≥40 years old in Japan is 12.4%; that is, approximately 8.1 million people suffer from OAB. OAB is strongly correlated with age and the incidence in elderly people (≥70 years old) exceeds 30% [4]. OAB reduces quality of life (QOL) and is a significant health problem [5].

The major treatment for OAB is pharmacotherapy, and anticholinergics (muscarinic receptor antagonists) are recommended as the first option. However, these drugs act on muscarinic receptors throughout the body and may cause adverse events such as dry mouth and constipation [6]. Imidafenacin and solifenacin were introduced in Japan in 2006 and 2007, respectively. Both drugs have excellent selectivity for the bladder over the salivary gland [7–11].

Several comparative trials of anticholinergics have been performed, but these have generally used a study period of up to 12 weeks [12–22]. Therefore, the long-term drug tolerability needs to be evaluated in an actual clinical situation because OAB is a chronic disease. It is important for clinicians to understand the pharmacological profiles of drugs for OAB and to choose a drug based on a long-term perspective. Therefore, we conducted a 52-week prospective randomized comparative study to evaluate the efficacy and tolerability of imidafenacin and solifenacin as novel anticholinergics for OAB. 

## 2. Methods

### 2.1. Ethics

This study was performed as a prospective, open, randomized, parallel-group trial to compare the long-term efficacy and tolerability of imidafenacin and solifenacin in Japanese patients with new OAB. The study was conducted in accordance with the Helsinki Declaration after approval of the Kanto Rosai Hospital Ethics Committee. Before enrollment in the study, the objectives and methods of the study and other necessary issues related to protection of human rights were explained to the patients. All patients gave written informed consent on a voluntary basis prior to enrollment in the study.

### 2.2. Subjects

Patients were enrolled in this study from January to December 2009 and followed up until December 2010. The subjects were male and female patients aged ≥50 and <80 years old who were diagnosed with OAB based on their overactive bladder symptom score (OABSS: 0–15 range, with a higher score indicating a severer condition) and had not been treated with any anticholinergics. Patients with a score for urinary urgency of ≥2 points and a total OABSS of ≥3 points were enrolled in the study. The other inclusion criteria were symptoms for at least 4 weeks and untreated OAB. The exclusion criteria were a history of pharmacotherapy of OAB; complications of diseases contraindicated for anticholinergics (serious heart disease, untreated angle-closure glaucoma, myasthenia gravis, gastric outlet and intestinal obstruction, paralytic ileus, and gastric and intestinal atony) residual urine volume ≥100 mL (determined by abdominal sonography); strong possibility of prostate and bladder cancer; acute active urinary tract infection; a patient judged not to be eligible by an investigator in charge.

### 2.3. Study Design

**Figure 1:**
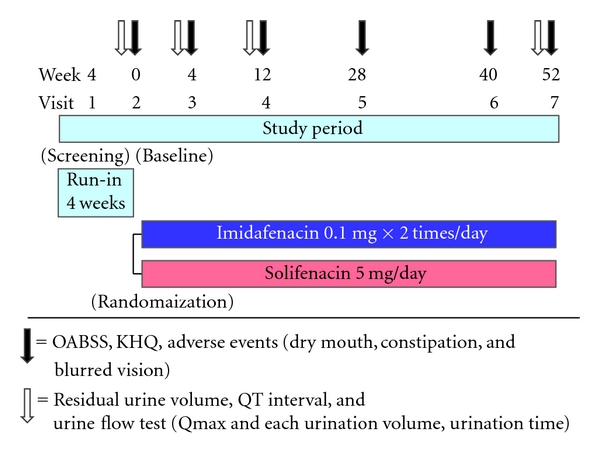
Design of the LIST study.

The study design is shown in Figure 1. Patients who gave informed consent were observed for 4 weeks to conduct tests for establishment of baseline data. Patients who met all the inclusion criteria and did not meet an exclusion criterion were randomly assigned to the imidafenacin or solifenacin group. Patients in the imidafenacin group took a 0.1 mg tablet of imidafenacin twice a day after breakfast and supper. Patients in the solifenacin group took a 5 mg tablet of solifenacin after breakfast. The administration period was scheduled to be 52 weeks and patients who took imidafenacin and solifenacin for at least 12 weeks were included in the efficacy analysis. Random assignment to groups was performed by the central registration system in the pharmacy, and age and sex were used as factors in the assignment.

Similar drugs for urinary frequency, urinary incontinence, and OAB anticholinergics (tolterodine, propiverine, oxybutynin, and flavoxate), cholinergics, and any drug under development for which the effects were not clarified were not allowed during the study period to avoid effects of concomitant drugs on the efficacy evaluation of the investigational drugs and for the safety of the subjects. An alpha-1 blocker was allowed as a concomitant drug with the limitation that a specific drug was used without a change in dosage and administration and was not replaced with another drug during the study period (observation and treatment phase). Urologic and genital surgery, catheterization, intermittent urethral catheterization, and electric and magnetic stimulation therapy were also restricted during the study period.

### 2.4. Evaluation of Efficacy and Tolerability

Age, sex, body weight, history of OAB, residual urine volume, urine flow test, prostate volume and electrocardiography (ECG) were evaluated at the start of the observation period as background data.

The primary endpoint for efficacy was the change in OABSS from baseline. The primary endpoints for safety were the 3 adverse events caused by anticholinergics: dry mouth, constipation, and blurred vision. The severity of dry mouth was evaluated on a 3-point scale: mild, barely noticeable; moderate, tolerable after drinking water; severe, intolerable after drinking water, leading to discontinuation of the investigational drug. The investigators determined whether a patient had constipation or blurred vision based on a face-to-face interview. Constipation was evaluated subjectively by the patients themselves and this information was obtained during consultation. Patients with constipation received oral drugs. Similarly to constipation, visual impairment was subjectively evaluated by patients themselves during visits for consultation.

The secondary endpoints for efficacy were changes in OABSS components from baseline (daytime frequency, nighttime frequency, urgency, and urgency incontinence) and the King's Health Questionnaire (KHQ) score. The KHQ is a QOL questionnaire that is specific for urinary incontinence and has been translated into several languages, including Japanese. The secondary endpoints for safety were adverse events, laboratory tests, blood pressure, pulse rate, urine flow test, residual urine volume, and 12-lead ECG (QT interval). The residual urine volume was determined by abdominal sonography immediately after urination. The prostate volume was calculated based on transabdominal ultrasound findings at the time of enrollment in the study.

Efficacy and safety were evaluated at 6 measurement points: at the end of the observation period, and 4, 12, 28, 40, and 52 weeks after the beginning of the treatment period. Residual urine volume, urine flow test (Qmax, urination volume, urination time), and 12-lead ECG were determined at the end of the observation period, and 4, 12, and 52 weeks after the beginning of the treatment period. The investigators conducted an interview using the OABSS and KHQ at every visit. Drug compliance was confirmed by the patients themselves at every visit. If a patient requested discontinuation of the drug during consultation, the investigator interviewed the patient to determine the reason. If a patient did not visit the hospital, the reason was determined by telephone.

### 2.5. Statistical Analysis

**(1) Efficacy Analysis**

The efficacy analysis set consisted of patients who took imidafenacin and solifenacin for at least 12 weeks, excluding ineligible patients and patients who received a restricted concomitant drug or therapy. Descriptive statistics for efficacy endpoints (values, amounts and rates of change) were calculated for each measurement point. Changes in the mean ± standard deviation (SD) or mean alone were plotted by group on a graph with the value on the *y*-axis and time on the *x*-axis. Descriptive statistics for values and changes in QOL score were calculated for each measurement point and changes (mean ± SD) from the end of the observation period are shown as a figure. Statistical analysis was performed by Wilcoxon test for intragroup comparison and by Mann-Whitney *U* test for intergroup comparison.

**(2) Safety Analysis**

The safety analysis set consisted of patients who took imidafenacin and solifenacin at least once and for whom information for safety was available after administration. The incidence of adverse events caused by anticholinergics (dry mouth, constipation, and blurred vision) was evaluated by Fisher Exact test and the severity of dry mouth was evaluated by Mann-Whitney *U* test. Kaplan-Meier curves for the time to the first adverse event caused by anticholinergics were evaluated by logrank test. Residual urine volume and QT interval were evaluated by Wilcoxon test. The significance level was set at 0.05.

## 3. Results

**Figure 2:**
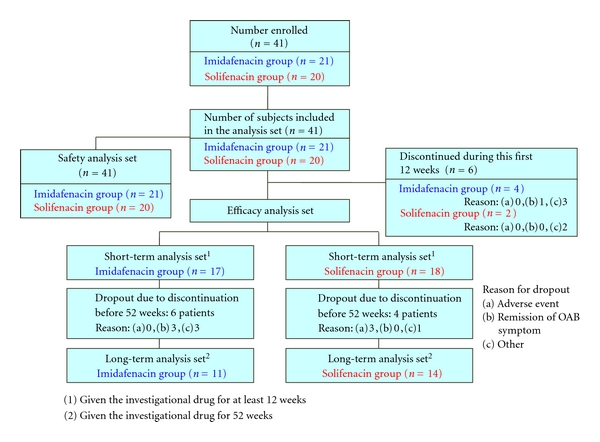
Summary of patient flow in the LIST study.

The details of the patients are shown in Figure 2. A total of 41 patients with untreated OAB who visited our hospital from January to December 2009 and gave informed consent were enrolled in the study, with 21 and 20 randomly assigned to the imidafenacin and solifenacin groups, respectively. Consequently, the safety analysis set consisted of 41 patients.

The short-term efficacy analysis set consisted of 35 patients (83.3%) who continuously took imidafenacin or solifenacin for at least 12 weeks, including 17 in the imidafenacin group and 18 in the solifenacin group. The long-term efficacy analysis set consisted of 25 patients (62.5%) who continuously took imidafenacin or solifenacin for 52 weeks, including 11 in the imidafenacin group and 14 in the solifenacin group. There was no significant difference in the rate of treatment completion between the two groups (*P* = 0.2477). Six patients (14.3%) in the imidafenacin group and 4 (9.5%) in the solifenacin group took the drug continuously for at least 12 weeks, but for less than 52 weeks. Among these patients, discontinuation occurred through patient choice due to remission of OAB symptoms in 3 (7.1%) in the imidafenacin group, and due to severe dry mouth in 1 (2.4%) and lack of efficacy in 2 (4.8%) in the solifenacin group. Other reasons for discontinuation were similar in the two groups.

The background of the 35 patients who took an investigational drug for at least 12 weeks and were included in the short-term efficacy analysis is shown in Table 1. There was no significant difference in age, sex, OABSS at baseline (8.0 ± 2.0 versus 8.7 ± 2.4), and percentage of cases with moderate severity (15/17, 88.2% versus 14/17, 77.8%) in the imidafenacin and solifenacin groups. The score for each of the 4 OABSS components also did not differ between the groups (*data not shown*). There was also no difference in residual urine volume, voiding volume, voiding time, maximum urine flow rate (Qmax), QT interval, prostate volume in male patients, and frequency of administration of alpha blockers between the groups.

The background of the 25 patients who took an investigational drug for 52 weeks and were included in the long-term efficacy analysis is shown in Table 2. Among these patients, there were no significant differences in background factors and OABSS at baseline (9.0 ± 1.3 versus 8.9 ± 2.4) in the imidafenacin and solifenacin groups.

### 3.1. Efficacy

**Figure 3:**
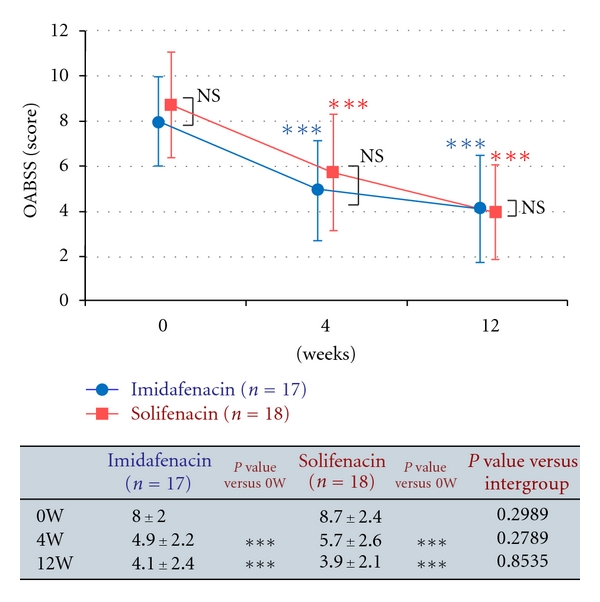
Changes in OABSS in the short-term analysis set. The endpoint is presented as a mean ± standard deviation. Intragroup comparison (versus the end of the observation period) by Wilcoxon test, ***P < 0.001. Intergroup comparison by Mann-Whitney *U* test, NS: not significant.

**Figure 4:**
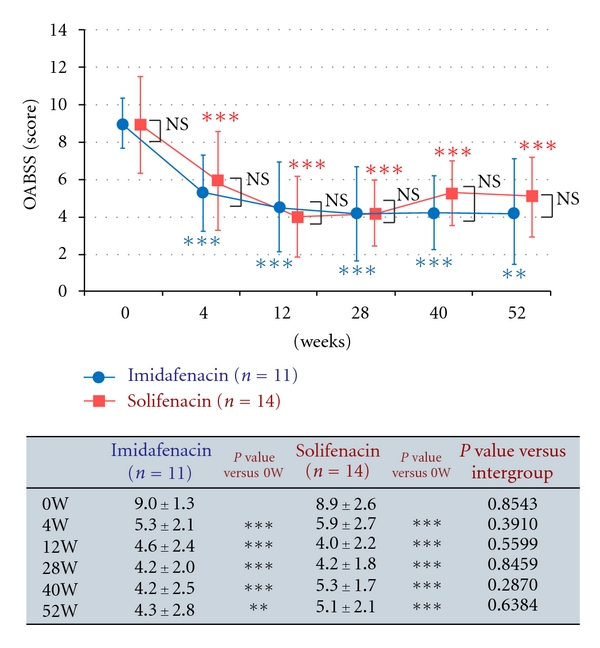
Changes in OABSS in the long-term analysis set. The endpoint is presented as a mean ± standard deviation. Intragroup comparison (versus the end of the observation period) by Wilcoxon test, **P < 0.01, ***P < 0.001. Intergroup comparison by Mann-Whitney *U* test, NS: not significant.

Efficacy over 12 weeks was evaluated in the short-term efficacy analysis set of 35 patients. Changes in the total OABSS in this set are shown in Figure 3. Changes in the total OABSS in the long-term efficacy analysis set of 25 patients are shown in Figure 4. The results of efficacy analysis showed no significant difference in efficacy between imidafenacin and solifenacin for treatment over 12 weeks and 52 weeks. With regard to changes in OABSS components from baseline, the score for the number of urination events at night at 52 weeks after the beginning of treatment changed by −0.6 ± 0.7 in the imidafenacin group and by 0.1 ± 0.8 in the solifenacin group, with a significant difference between the groups. No other OABSS component scores differed significantly between the two groups at any measurement point (*data not shown*).

**Figure 5:**
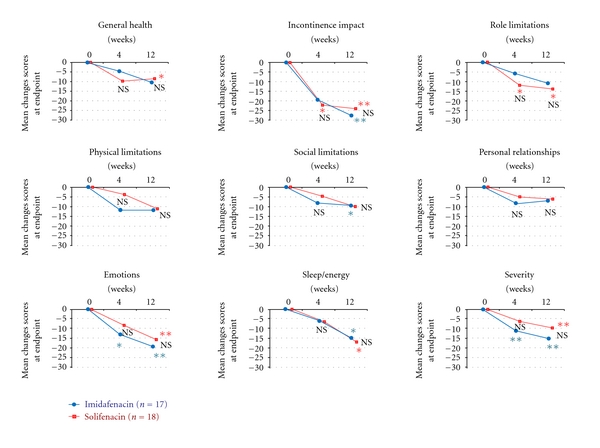
Mean changes in KHQ domain scores from baseline to endpoint in the short-term analysis set. The endpoint is presented as a mean. Intergroup comparison by Mann-Whitney *U* test, NS: not significant. Intragroup comparison (versus the end of the observation period) by Wilcoxon test, *P < 0.05, **P < 0.01.

**Figure 6:**
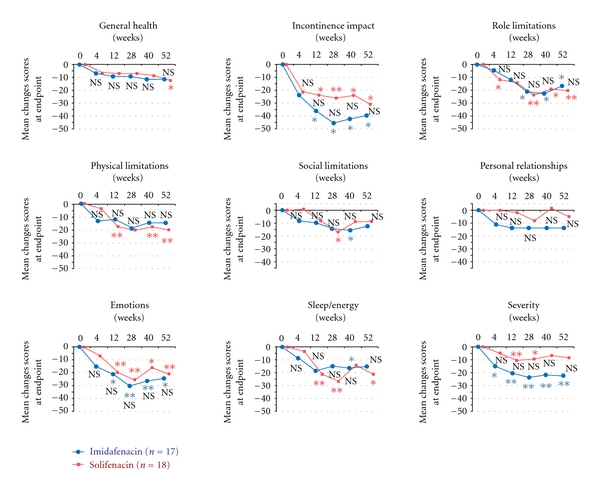
Mean changes in KHQ domain scores from baseline to endpoint in the long-term analysis set. The endpoint is presented as a mean. Intergroup comparison by Mann-Whitney *U* test, NS: not significant. Intragroup comparison (versus the end of the observation period) by Wilcoxon test, *P < 0.05, **P < 0.01.

Changes in scores for KHQ domains relative to those at baseline in the 35 patients in the short-term efficacy set and the 25 patients in the long-term efficacy set are shown in Figures 5 and 6, respectively. Changes in scores for domains on the KHQ (a QOL questionnaire specific for urinary incontinence) from baseline did not differ between the two groups at 12 and 52 weeks. Thus, the results of this study showed that imidafenacin and solifenacin had similar efficacy for OAB for both a short period of 12 weeks and a long period of 52 weeks.

### 3.2. Safety and Tolerability

**Figure 7:**
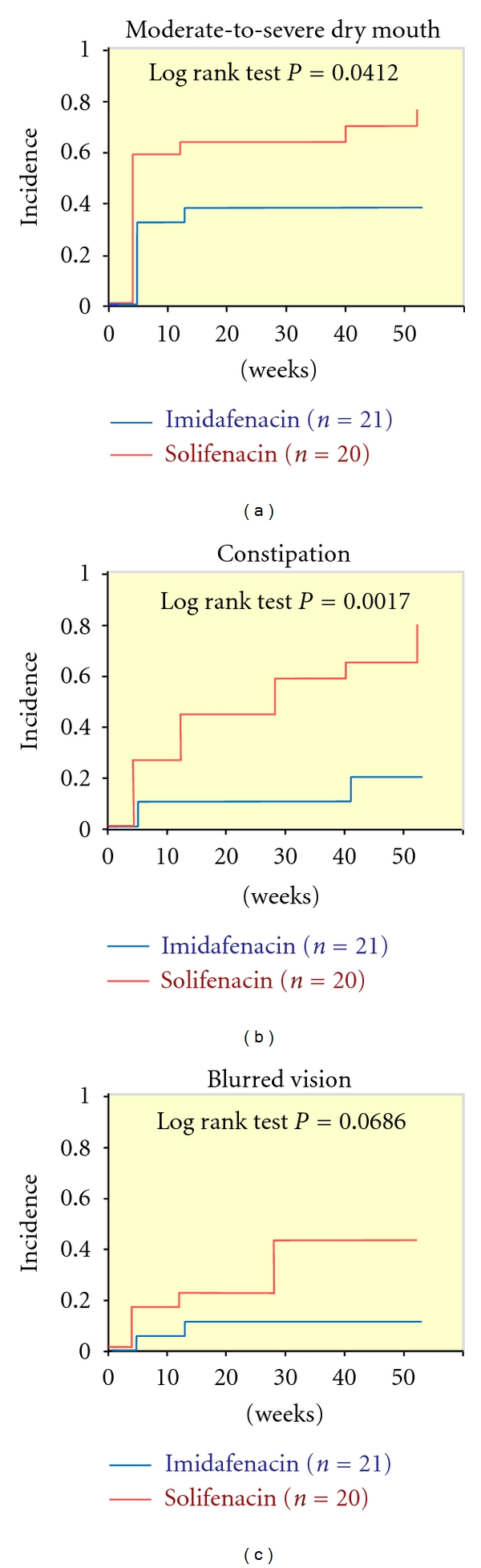
Kaplan-Meier curves for the time to the first adverse event caused by the anticholinergic agent (52 weeks).

The tolerability of imidafenacin and solifenacin was good. Adverse events occurred in 76.2% of patients in the imidafenacin group and 95.0% in the solifenacin group. One patient discontinued solifenacin due to severe dry mouth whereas there was no case of discontinuation of imidafenacin for this reason. The incidence of dry mouth over 52 weeks was 71.4% in the imidafenacin group and 90% in the solifenacin group, with no significant difference between the groups. However, severe dry mouth was observed in 4 patients (20%) in the solifenacin group, but in no patients in the imidafenacin group. Furthermore, the cases of dry mouth in the imidafenacin group were significantly milder than those in the solifenacin group (Mann-Whitney *U* test: *P* = 0.0092). The overall severity of dry mouth was also significantly milder in the imidafenacin group throughout the study period (Table 3). The incidence of constipation over 52 weeks was also significantly lower in the imidafenacin group (14.3% versus 65.0%, Table 3). The incidence of blurred vision for 52 weeks did not differ significantly between the groups (9.5% versus 35.0%, Table 3) and there were no adverse events related to blood pressure and pulse rate in the two groups. Kaplan-Meier curves for the time to the first adverse event caused by anticholinergics are shown in Figure 7. There was no difference in the incidence of moderate or severe dry mouth between the groups in the 12-week analysis (logrank test: *P* = 0.0616, *data not shown*), but this incidence differed significantly in the 52-week analysis (Log Rank test: *P* = 0.0412; Figure 7). There was also no difference in the incidence of constipation between the two groups in patients who took imidafenacin or solifenacin for at least 12 weeks (Log Rank test: *P* = 0.0621, data not shown). However, the incidence in patients who took either drug for 52 weeks was significantly higher in the solifenacin group (Log Rank test: *P* = 0.0017). There was no significant difference in the incidence of blurred vision in patients who took the drug for at least 12 weeks (Log Rank test: *P* = 0.3749, data not shown) or continuously for 52 weeks (Log Rank test: *P* = 0.0686).

Changes in the residual urine volume and QT interval are shown in Tables 4 and 5, respectively. Significant increases in the residual urine volume occurred in both groups at 12 weeks compared to baseline. No increase in the residual urine volume from baseline occurred in the imidafenacin group at 52 weeks, but a significant increase was found in the solifenacin group. There was no significant difference between the two groups, and no patient had more than 100 mL of residual urine volume in either group. No significant change in QT interval was observed throughout the study period in either group. Furthermore, although the results of urine flow tests (Qmax, urination volume, and urination time) slightly changed in both groups, no significant changes were found after 12 and 52 weeks.

## 4. Discussion

This study is the first prospective randomized comparative study of the efficacy and tolerability of anticholinergics for a period of 52 weeks in patients with OAB. Previous comparative studies of anticholinergics have used a study period of up to 12 weeks [12–22], but OAB is a chronic disease and there is a need to examine the long-term efficacy and tolerability of drugs used to treat OAB. The results of this study suggest that anticholinergics should be chosen based on long-term efficacy and safety. As expected, we found no significant difference in OABSS and KHQ scores between the imidafenacin and solifenacin groups. The incidence of dry mouth in the 12-week analysis was 16.9% whereas that in the 52-week analysis was 39.2%, which emphasizes the importance of evaluating long-term safety. The severity of dry mouth was milder at a dose of 0.2 mg/day imidafenacin than at 5 mg/day solifenacin, and intergroup differences in the severity of dry mouth increased with time. 

Imidafenacin and solifenacin are novel anticholinergics for OAB that were designed to have excellent selectivities for the bladder over the salivary gland and for the M3 receptor that controls contraction of bladder smooth muscle. The efficacy and tolerability of imidafenacin (0.2 mg/day) and solifenacin (5 mg and 10 mg/day) were shown in a 12-week phase III double blind comparative trial performed in Japan with propiverine (20 mg/day) as a control [20, 22]. With regard to safety, the incidence and severity of dry mouth was significantly lower at a dose of 0.2 mg/day imidafenacin than at 20 mg/day propiverine, and the incidence of constipation was also lower. The incidence of dry mouth was also significantly lower at 5 mg/day solifenacin, but significantly higher at 10 mg/day solifenacin, in comparison with that at 20 mg/day propiverine; and the incidence of constipation was significantly higher at 10 mg/day solifenacin than at 20 mg/day propiverine. Consequently, the regular dose of solifenacin was set at 5 mg/day.

Based on these previous reports, 0.2 mg/day imidafenacin and 5 mg/day solifenacin were used as the doses in this study. This choice was made for several reasons. First, most patients are treated at a low initial dose in practice and 20% to 30% subsequently require an increased dose. Second, there is a possibility that the frequency of the increase in dose could differ between the two groups, if an increase in dose is allowed. Third, comparison of a low dose with less adverse events is reasonable in evaluation of long-term tolerability. 

In a 12-week phase III trial in Japan, the incidence of constipation with 5 mg/day solifenacin was 10.6%, and did not differ from that with 0.2 mg/day imidafenacin or 20 mg/day propiverine [22]. In 52-week administration, the incidences were 9.9% and 19.0% with imidafenacin [23] and 5 mg/day solifenacin [24], respectively; that is, approximately double those in the phase III trial. These results suggest that constipation is an adverse event of anticholinergics of similar importance to dry mouth. Meek et al. recently conducted meta-analysis on the relationship between constipation and anticholinergics in OAB patients and found that the risk for constipation doubled in patients treated with anticholinergics for at least 2 weeks. The odds ratios were 3.0 for solifenacin, 2.9 for trospium, 2.3 for oxybutynin, 2.1 for fesoterodine, 1.9 for darifenacin, and 1.4 for tolterodine. Thus, the risk for constipation differed between drugs, and this variation may depend on differences in affinity for muscarinic receptors among the drugs [25].

The different incidences of adverse events with imidafenacin and solifenacin might be due to differences in pharmacokinetics between the two drugs. In mouse and rat, Yamada et al. found that anticholinergics had different organ-specific affinity for muscarinic receptors [26–29]. Solifenacin has a long half-life and binds to muscarinic receptors in not only the bladder but also the salivary gland and colon for an extended period [26], which reduces salivation and intestinal peristaltic movement and results in severe dry mouth and constipation. In contrast, imidafenacin has a short half-life and does not bind to muscarinic receptors in the colon [29], accounting for the low incidence of constipation. However, imidafenacin binds to muscarinic receptors in the bladder for a relatively long time, despite the short blood half-life [29]. 

A limitation of this study is the small-scale single-center design. However, conducting the study at a single institution also excludes interobserver variability and variation between institutions, and this improves the reliability of the results. We emphasize that this clinical trial was performed under conditions seen in actual clinical practice. In daily practice, unlike in trials of new drug development, a physician must respect a patient's request and this leads to frequent changes in treatment plans. The reasons for drug discontinuation were surveyed by telephone or interview, and we found that the persistence of drug use in the study was good compared to previous reports of actual clinical practice. Thus, Wagg et al. found that the persistence was relatively low for most antimuscarinics after 12 months (27.1–36.3%, including oxybutynin, solifenacin, tolterodine, and trospium) [30]. There were 10 dropout patients in the current study, since 35 patients took either drug for at least 12 weeks and were included in the efficacy analysis set, but only 25 of these patients took a drug continuously for 52 weeks (Figure 2). Of these 10 patients, 6 were in the imidafenacin group and 4 in the solifenacin group. However, the reason for discontinuation was remission of OAB symptoms in 3 patients in the imidafenacin group, reflecting a positive effect of the drug. The other 3 patients in this group discontinued the trial due to onset of neurogenic bladder caused by cerebral infarction (a causal relationship between the onset of cerebral infarction and imidafenacin was excluded) in 1 case and frequent visits for another disease in 2 cases; thus, none of the patients who discontinued imidafenacin did so for negative reasons associated with the drug. In contrast, 2 patients discontinued solifenacin due to the absence of an effect and one patient discontinued due to a severe adverse reaction to solifenacin; thus, 3 patients discontinued solifenacin for negative reasons. The 6 patients who took imidafenacin or solifenacin for less than 12 weeks and were not included in the efficacy analysis did not discontinue for negative reasons. There was no difference in the percentage of patients who received continuous treatment in the two groups, and these data rule out the possibility that the apparent efficacy in the 52-week analysis was due to dropout of patients in whom the drugs were ineffective. The possibility that dropout patients influenced the results of the long-term analysis was also ruled out because dropout for adverse events occurred only in the solifenacin group.

Imidafenacin and solifenacin were both developed to reduce adverse events caused by anticholinergics. The results of this study indicate that long-term tolerability to imidafenacin is superior to that for solifenacin while there was no difference in efficacy between the two groups. We note that the doses of both drugs can be increased, and therefore further studies should be conducted to compare long-term tolerability at higher doses. Drugs with novel mechanisms, including *β*3 receptor agonists, are also currently under development for treatment of OAB, and trials to evaluate the long-term efficacy and tolerability of these drugs will be important since OAB is a QOL-related disease.

## 5. Conclusions

This study is the first long-term trial to show differences in the properties of anticholinergics that could not be detected in short-term studies. Imidafenacin and solifenacin were both effective for OAB, but the incidence of adverse events with imidafenacin was significantly lower than that with solifenacin. There were also time-dependent differences in the severity and incidence of adverse events. Since OAB is a chronic disease, we conclude that treatment with imidafenacin is preferable to solifenacin from a perspective of safety.

## Figures and Tables

**Table 1:** Baseline characteristics of subjects for short-term analysis set.

Demographics		Imidafenacin	Solifenacin	*P*-value
Subjects		17	18	
Age (years)	(Mean ± SD)	70.2 ± 6.5	69.8 ± 7.7	0.8697^a^
<60, *n *(%)		0 (0%)	1 (5.6%)	
≥60, *n *(%)		8 (47.1%)	6 (33.3%)	
≥70, *n* (%)		9 (52.9%)	11 (61.1%)	
Gender				
Male, *n *(%)		10 (58.8%)	12 (66.7%)	0.7332^b^
Female, *n *(%)		7 (41.2%)	6 (33.3%)	
OABSS (total score)	(Mean ± SD)	8.0 ± 2.0	8.7 ± 2.4	0.2989^c^
Severity of OABSS				
Mild, *n *(%)		2 (11.8%)	3 (16.7%)	1.0000^c^
Moderate, *n *(%)		15 (88.2%)	14 (77.8%)	
Severe, *n *(%)		0 (0%)	1 (5.6%)	
Postvoid residual volume (mL)	(Mean ± SD)	14.2 ± 15.1	13.5 ± 11.4	0.8814^a^
Each urination volume (mL)	(Mean ± SD)	141.7 ± 109.9	187.9 ± 160.8	0.3312^a^
Urination time (second)	(Mean ± SD)	28.3 ± 19.8	27.4 ± 9.9	0.8749^a^
Qmax (mL/s)	(Mean ± SD)	12.1 ± 7.4	14.2 ± 10.6	0.4925^a^
In male				
Prostate volume (mL)	(Mean ± SD)	22.7 ± 11.6	25.3 ± 9.4	0.5661^a^
Use of *α*1 blocker, *n *(%)		9 (90%)	9 (75%)	0.8328^b^

Severity of OAB was defined as total OABSS score ≤5: mild; 6 to ≤11: moderate; ≥12: severe

a: Unpaired *t*-test, b: Fisher's exact test, c: Mann-Whitney *U* test, two-sided. SD: standard deviation.

**Table 2:** Baseline characteristics of subjects for long-term analysis set.

Demographics		Imidafenacin	Solifenacin	*P*-value
Subjects		11	14	
Age (years)	(Mean ± SD)	69.9 ± 6.7	71.4 ± 6.0	0.5560^a^
<60, *n *(%)		0 (0%)	0 (0%)	
≥60, *n *(%)		4 (36.4%)	4 (28.6%)	
≥70, *n *(%)		7 (63.6%)	10 (71.4%)	
Gender				
Male, *n *(%)		7 (63.6%)	10 (71.4%)	1.0000^b^
Female, *n *(%)		4 (36.4%)	4 (28.6%)	
OABSS (total score)	(Mean ± SD)	9.0 ± 1.3	8.9 ± 2.6	0.8453^c^
Siverity of OABSS				
Mild, *n *(%)		0 (0%)	3 (21.4%)	0.3671^c^
Moderate, *n *(%)		11 (100%)	10 (71.4%)	
Severe, *n *(%)		0 (0%)	1 (7.1%)	
Postvoid residual volume (mL)	(Mean ± SD)	15.1 ± 14.7	13.4 ± 11.0	0.7487^a^
Each urination volume (mL)	(Mean ± SD)	125.3 ± 85.0	203.8 ± 179.8	0.1654^a^
Urination time (second)	(Mean ± SD)	29.1 ± 23.0	26.1 ± 10.7	0.6939^a^
Qmax (mL/s)	(Mean ± SD)	11.2 ± 8.8	14.8 ± 11.8	0.4120^a^
In male				
Prostate volume (mL)	(Mean ± SD)	21.0 ± 9.1	27.8 ± 8.2	0.1302^a^
Use of *α*1 blocker, *n *(%)		6 (85.7%)	9 (90.0%)	1.0000^b^

Severity of OAB was defined as total OABSS score ≤5: mild; 6 to ≤11: moderate; ≥12: severe

a: Unpaired *t*-test, b: Fisher's exact test, c: Mann-Whitney *U* test, two-sided. SD: standard deviation.

**Table 3:** Distribution of major anticholinergic adverse events in each group over 52 weeks.

Variable, *n *(%)	Imidafenacin	Solifenacin	*P*-value
*N* (safety population)	21	20	
Dry mouth	15 (71.4%)	18 (90.0%)	0.2379^b^
Mild	8 (38.1%)	4 (20.0%)	
Moderate	7 (33.3%)	10 (50.0%)	0.0092^a^
Severe	0 (0%)	4 (20.0%)	
Constipation	3 (14.3%)	13 (65.0%)	0.0013^b^
Blurred vision	2 (9.5 %)	7 (35.0%)	0.0670^b^

The severity of dry mouth was evaluated on a 3-point scale: mild, barely noticeable; moderate, tolerable after drinking water; severe, intolerable after drinking water, leading to discontinuation of the investigational drug;

a: Mann-Whitney *U* test, b: Fisher exact test, two-sided.

**Table 4:** Changes in residual urine volume and QT interval in the short-term analysis set.

Variable		Imidafenacin	Solifenacin	
Number of subjects		17	18	
Residual urine volume (mL)				
0 week	(Mean ± SD)	14.2 ± 15.1	13.5 ± 11.4	
4 week	(Mean ± SD)	29.3 ± 26.2**	40.8 ± 33.2**	NS
12 week	(Mean ± SD)	37.7 ± 32.7**	29.4 ± 28.1*	NS
QT interval (ms)				
0 week	(Mean ± SD)	414.8 ± 20.7	423.2 ± 21.8	
4 week	(Mean ± SD)	415.5 ± 16.5	423.5 ± 19.0	NS
12 week	(Mean ± SD)	415.0 ± 16.4	423.6 ± 23.5	NS

Intragroup (versus 0 weeks: the end of the observation period)

Paired *t*-test *: *P* < 0.05, **: *P* < 0.01

Intergroup: unpaired *t*-test, NS: not significant, SD: standard deviation.

**Table 5:** Changes in residual urine volume and QT interval in the long-term analysis set.

Variable		Imidafenacin	Solifenacin	
Number of subjects		11	14	
Residual urine volume (mL)				
0 week	(Mean ± SD)	15.1 ± 14.7	13.4 ± 11.0	
4 week	(Mean ± SD)	30.9 ± 20.4*	38.6 ± 32.1**	NS
12 week	(Mean ± SD)	30.7 ± 28.0	30.6 ± 27.7*	NS
52 week	(Mean ± SD)	20.6 ± 25.4	31.1 ± 23.4**	NS
QT interval (ms)				
0 week	(Mean ± SD)	413.6 ± 24.6	426.9 ± 22.4	
4 week	(Mean ± SD)	415.8 ± 18.9	426.2 ± 20.1	NS
12 week	(Mean ± SD)	416.4 ± 18.4	427.2 ± 23.0	NS
52 week	(Mean ± SD)	417.0 ± 20.3	424.4 ± 22.5	NS

Intragroup (versus 0 weeks: the end of the observation period)

Paired *t*-test *: *P* < 0.05, **: *P* < 0.01

Intergroup: Unpaired *t*-test, NS: not significant, SD: standard deviation.

**Table 6:** Overactive Bladder Symptom Score (OABSS)*.

Question	Frequency	Score
How many times do you typically urinate from waking in the morning until sleeping at night?	≤7	0
	8–14	1
	≥15	2
How many times do you typically wake up to urinate from sleeping at night until waking in the morning?	0	0
	1	1
	2	2
	≥3	3
How often do you have a sudden desire to urinate, which is difficult to defer?	Not at all	0
	Less than once a week	1
	Once a week or more	2
	About once a day	3
	2–4 times a day	4
	5 times a day or more	5
How often do you leak urine because you cannot defer the sudden desire to urinate?	Not at all	0
	Less than once a week	1
	Once a week or more	2
	About once a day	3
	2–4 times a day	4
	5 times a day or more	5

*Patients were instructed to circle the score that best applied to their urinary condition during the past week; the overall score was the sum of the four scores.

**Table 7:** The King's Health Questionnaire (KHQ).

KHQ question	Significant content
(1) How would you evaluate your health today?	Health
(2) How much do you think your bladder problem hampers your life?	Bladder problems, hampering your life
(3) Frequency: do you go the bathroom too often?	Frequency going to the bathroom
(4) Nocturia: do you get up at night to urinate?	Nocturia, getting up
(5) Urgency: do you feel urgency to urinate and have difficulty controlling it?	Urinary urgency
(6) Hyperactive bladder: do you lose urine when you feel urgency to urinate?	Hyperactive bladder
(7) Urinary incontinence by exertion: do you lose urine during physical activities?	Urinary incontinence, physical activities
(8) Nocturnal enuresis: do you wet your bed at night?	Nocturnal enuresis
(9) Incontinence during sexual intercourse: do you lose urine during sexual intercourse?	Incontinence during sexual intercourse
(10) Frequent infections: do you have many urinary infections?	Urinary infections
(11) Pain in the bladder: do you feel pain in the bladder?	Pain in the bladder
(12) Do you have any other problem related to your bladder?	Problem related to your bladder
(13) How intensely do your bladder problems hinder your house chores? (cleaning, washing, and cooking, etc.)	House chores (cleaning, washing, cooking, etc.)
(14) How intensely do your bladder problems hinder your work or your daily activities outside the house? (shopping, taking children to school, etc.)	Work, daily activities outside the house (shopping, taking children to school, etc.)
(15) Do your bladder problems hinder your physical activities? (walking, running, or any other sport?)	Physical activities (walking, running, or any other sport)
(16) Do your bladder problems hinder you when you want to travel?	Travelling
(17) Do your bladder problems hinder you when go to the church, a meeting, a party?	Church, meeting, and party
(18) Do you avoid visiting friends because of your bladder problems?	Visiting friends
(19) Do your bladder problems hinder your sexual life?	Sex life
(20) Do your bladder problems hinder your life with your partner/husband?	Partner/husband
(21) Do your bladder problems disturb your family members?	Disturbing family members
(22) Do you feel depressed with your bladder problems?	Depression
(23) Do you feel anxious or nervous with your bladder problems?	Anxious, nervous
(24) Do you feel bad about yourself because of your bladder problems?	Feeling bad about yourself
(25) Do your bladder problem hinder your sleep?	Sleep
(26) Do you use any kind of hygienic protection such as diapers, pads, or lining to keep yourself dry?	Hygienic protection
(27) Do you control the amount of liquids you drink?	Controlling the amount of liquids, drinking
(28) Do you need to change your underwear (panties) when you get wet?	Changing underwear
(29) Do you worry about smelling like urine?	Smelling of urine

## Appendix

See Tables 6 and 7, [31, 32].

## Abbreviations

OAB: Overactive bladder
OABSS: Overactive Bladder Symptom Score
KHQ: King's Health Questionnaire.
